# A Comparative Oncology Study of Iniparib Defines Its Pharmacokinetic Profile and Biological Activity in a Naturally-Occurring Canine Cancer Model

**DOI:** 10.1371/journal.pone.0149194

**Published:** 2016-02-11

**Authors:** Corey Saba, Melissa Paoloni, Christina Mazcko, William Kisseberth, Jenna H. Burton, Annette Smith, Heather Wilson-Robles, Sara Allstadt, David Vail, Carolyn Henry, Susan Lana, E. J. Ehrhart, Brad Charles, Michael Kent, Jessica Lawrence, Kristine Burgess, Antonella Borgatti, Steve Suter, Paul Woods, Ira Gordon, Patricia Vrignaud, Chand Khanna, Amy K. LeBlanc

**Affiliations:** 1 College of Veterinary Medicine, University of Georgia, Athens, Georgia, United States of America; 2 Comparative Oncology Program, Center for Cancer Research, National Cancer Institute, Bethesda, Maryland, United States of America; 3 College of Veterinary Medicine, The Ohio State University, Columbus, Ohio, United States of America; 4 College of Veterinary Medicine and Biological Sciences, Colorado State University, Fort Collins, Colorado, United States of America; 5 College of Veterinary Medicine, Auburn University, Auburn, Alabama, United States of America; 6 College of Veterinary Medicine, Texas A&M University, College Station, Texas, United States of America; 7 School of Veterinary Medicine, University of California Davis, Davis, California, United States of America; 8 School of Veterinary Medicine, University of Wisconsin-Madison, Madison, Wisconsin, United States of America; 9 College of Veterinary Medicine, University of Missouri-Columbia, Columbia, Missouri, United States of America; 10 School of Veterinary Medicine, Tufts University, North Grafton, Massachusetts, United States of America; 11 College of Veterinary Medicine, University of Minnesota, St. Paul, Minnesota, United States of America; 12 College of Veterinary Medicine, North Carolina State University, Raleigh, North Carolina, United States of America; 13 Ontario Veterinary College, University of Guelph, Guelph, Ontario, Canada; 14 Sanofi Aventis, Guesde, Vitry-sur-Seine, 94403 Cedex, France; Cornell University, UNITED STATES

## Abstract

Development of iniparib as an anti-cancer agent was hindered in part by lingering questions regarding its mechanism of action, the activity of its metabolites, and their potential accumulation in tumors. Due to strong similarities in metabolism of iniparib between humans and dogs, a veterinary clinical trial in pet dogs with spontaneous cancers was designed to answer specific questions pertaining to pharmacokinetic exposures and tolerability of iniparib. Dogs were treated with iniparib alone and in combination with carboplatin chemotherapy. Iniparib doses ranged between 10–70 mg/kg intravenously (IV). Plasma, tumor and normal tissue samples were collected before and at various time points scheduled after exposure for pharmacokinetic and biologic analysis. The primary endpoints included characterization of dose-limiting toxicities (DLT) and determination of the drug exposures that could be achieved in both normal and tumor tissues. Nineteen dogs were treated. DLT included fever, anorexia, diarrhea, neutropenia, and thrombocytopenia; most effects were attributable to carboplatin based on the timing of adverse event onset. The maximum tolerated dose (MTD) of iniparib was not identified. Moderate to high variability in plasma exposure was noted for iniparib and all metabolites between animals. When quantifiable, iniparib and metabolite plasma:tumor ratios were < 0.088 and <1.7, respectively. In this study, iniparib was well tolerated as a single agent and in combination with carboplatin over a range of doses. However, clinically relevant concentrations of the parent drug and selected metabolites were not detectable in canine tumor tissues at any studied dose, thus eliminating expectations for clinical responses in dogs or humans. Negative clinical trials in humans, and the uncertainties of its mechanism of action, ultimately led to the decision to stop clinical development of the drug. Nevertheless, the questions that can be asked and answered within the comparative oncology approach are evident from this successfully executed comparative clinical trial and exemplify the value of such studies in drug development.

## Introduction

Iniparib (4-iodo-3-nitrobenzamide; BSI-201) is a highly lipophilic small-molecule prodrug that is metabolized to an active C-nitroso intermediate. Known downstream metabolites from this active intermediate include INBA (4-iodo-3-nitrosobenzamide), and IABM (4-iodo-3-amino-benzamide), but their distinct anti-cancer potencies are unknown. The proposed detoxification pathway of iniparib metabolism results in the production of several distinct conjugates and IABA (4-iodo-3-amino-benzoic acid). Iniparib was initially developed as a poly adenosine diphosphate ribose polymerase 1 (PARP1) inhibitor; however, current knowledge indicates that its activity is not linked to PARP inhibition as originally thought.[1,2] The integration of iniparib into cancer treatment protocols was originally supported by encouraging results from initial human clinical trials, specifically in the treatment of metastatic triple-negative breast cancer (TNBC).[3] However, a later stage, phase III study did not meet its primary endpoints of demonstrating an improvement in progression-free and overall survival. Although the clinical development of iniparib was ultimately stopped, publication of clinical studies of iniparib in combination with a variety of chemotherapy agents continues.[4, 5, 6]

Questions regarding iniparib’s mechanism of action, its metabolites, and their accumulation and biological activity in tumors and normal tissues remain unanswered. Differences in the metabolism of iniparib between humans and mice have precluded the use of murine models to investigate this drug, underscoring the need for alternative models to investigate the potential use of iniparib in cancer treatment protocols. When comparing studies in human patients and healthy beagle dogs, it was apparent that similarities in iniparib pharmacokinetics (PK) between these species exist, suggesting that dogs may be an important model for studying the true mechanisms of action, activity, and appropriate use of this drug. Specifically, doses of 10–30 mg/kg in research dogs have produced exposures of drug and metabolites comparable to the tested human clinical dose of 5.6 mg/kg (G. T. Emmons, personal communication). Plasma elimination (t1/2) of iniparib is rapid (< 10 to 20 minutes) while that of the IABM and IABA metabolites is slower (1–2 hours), in both species.

Similarities in iniparib tolerability between human patients and healthy beagles have been noted as well. When used as a single agent in human patients, toxicity has been minimal, and safety analyses indicate that the addition of iniparib to standard chemotherapy protocols does not appreciably add to their toxicity profiles. Single and multiple dose toxicokinetic studies of iniparib alone in normal beagle dogs have illustrated comparable tolerability at translatable doses above the human patient regimen (5.6 mg/kg IV twice weekly, see S1 Poster). Although these studies in human patients and healthy beagles have included few subjects, the data support translatable PK across species and further allow same-species therapeutic index questions to be asked. Collectively, this approach lends valuable support to the comparative approach to cancer drug development.

The comparative oncology approach, specifically referring to the study of pet dogs with spontaneous cancer, offers a potential solution to the lingering questions about iniparib, its metabolites, and their accumulation in tumors. In addition to the similarities in drug metabolism, the large size of pet dogs and their naturally-occurring, biologically heterogeneous malignancies allow for the collection of repeated blood and tissue samples to study drug PK and biodistribution. Such critical questions, when answered by the tumor-bearing dog model, add value to the current approaches during drug development. Therefore, a clinical trial in tumor-bearing dogs was designed with the following objectives: 1) to determine plasma and tumor exposures of iniparib and its metabolites, linked to tolerable iniparib doses, 2) to determine the therapeutic index (dose-biologic response and dose-toxicity) of iniparib, 3) to determine iniparib exposure in normal tissues, and 4) to define tumor iniparib and metabolite levels when used as a single agent and in combination with carboplatin. The long-term goal is to further validate this canine comparative oncology model in the development of new anti-cancer agents.

## Materials and Methods

### Comparative Oncology Trials Consortium (COTC): Clinical Trial Conduct

All dogs were evaluated uniformly and treated within a defined clinical protocol with Institutional Animal Care and Use Committee (IACUC) approval at each COTC enrollment site (The Ohio State University, Colorado State University, Auburn University, Texas A&M University, University of California-Davis, University of Missouri, and University of Wisconsin-Madison). The NCI-COP reviewed the eligibility screening and approved trial entry of each patient. Informed owner consent was required for trial entry. The goals, infrastructure, and data reporting of the COTC have been described previously.[7,8] Additionally, a Data/Safety Monitoring Board (DSMB) is convened for each COTC trial, comprised of 5 COTC investigators (1 chair and 4 committee members) who are not directly participating in the trial. This committee reviews all adverse events (AEs) and data generated from the trial on both a scheduled and ad-hoc basis depending on the severity and nature of the AEs. Decisions regarding AE management, dose modification/escalation, and modification of the clinical protocol are made jointly between the DSMB and Comparative Oncology Program personnel. This veterinary clinical trial in dogs with cancer described herein was conducted through this multi-institutional consortium. The study protocol was reviewed and approved by each participating institution’s Institutional Animal Care and Use Committee.

### Trial eligibility and enrollment

Client-owned pet dogs weighing > 10 kg with histologically confirmed malignant melanoma, squamous cell carcinoma, or soft tissue sarcoma were eligible for inclusion in this open-label prospective clinical trial. Other eligibility criteria required that dogs be free of significant co-morbid illness with a favorable performance status (grade 0 or 1 modified Eastern Cooperative Oncology Group (ECOG) performance status), have a measureable tumor(s) ≥ 3 cm at the longest diameter amenable to serial biopsy, and signed owner consent. Dogs with naïve, recurrent after previous surgery, or metastatic disease were eligible; however dogs receiving prior treatment with chemotherapy, immunotherapy, or radiation therapy were excluded. A 7-day washout period for non-steroidal anti-inflammatory drugs and corticosteroids was required prior to trial initiation.

Dogs underwent enrollment evaluation including measurement of tumor burden using calipers, digital photography of tumor burden, complete blood count (CBC), serum biochemical profile, urinalysis (UA), thoracic radiographs, and abdominal ultrasound examination (if clinically indicated) within 10 days of the first iniparib treatment (Day 1). Dogs were ineligible for the trial enrollment if the serum creatinine concentration > upper limits of normal, total serum bilirubin concentration > 2.0 mg/dL, hematocrit < 25%, platelet count < 50,000/μL, or any grade 2 or higher hematologic and/or biochemical abnormality as assessed by VCOG-CTCAE v1.1. [9]

### Study schema

Anticipated trial duration for each enrolled dog was 22 days. A schedule of patient evaluation and diagnostics performed at each time point is summarized in Table 1. Dogs were considered off study after completion of Day 22 and were free to receive any additional therapy deemed appropriate by the attending clinician. If disease progression and/or symptomatic deterioration were documented during the trial period, dogs were removed from the study, and Day 22 collections were performed early.

**Table 1:** Schedule of patient evaluations and study procedures.

Action	Pre-Treatment	Day 1	Day 4	Day 8	Day 11	Day 15	Day 22
Patient Eligibility	X						
Measurement of tumor burden (caliper measurements)	X			X		X	X
Physical Exam	X	X	X	X	X	X	X
Chest radiographs	X						
Digital photo of tumor	X			X		X	X
CBC/chemistry/UA/coagulation profile		X		X		X	X
Serum and plasma collection	X	X	X	X	X	X	X
Iniparib administration (IV over 60 minutes)		X	X	X	X		
Carboplatin administration				X			
Tumor Biopsy	X	X		X		X	
Normal tissue Biopsy	X	X		X		X	
Owner Assessment Form		X	X	X	X	X	X
Blood pressure measurement		X		X			

All tumor measurements were recorded in centimeters and performed independently by 2 clinicians. Although clinical activity was not a study endpoint, tumor measurements were compared between visits and responses determined via RECIST 1.1 criteria. [10] Hematologic and biochemical evaluation (CBC, serum biochemistry profile, coagulation parameters (PT/PTT), and UA) were performed at a central laboratory (Antech GLP, Morrisville, NC, USA) under GLP conditions. On Day 8, an additional CBC, blood urea nitrogen (BUN), and serum creatinine were performed at the respective COTC institution to ensure safe iniparib/carboplatin administration and to evaluate toxicities associated with administration of iniparib alone. Blood pressures (indirect or mean arterial pressure) were recorded on Days 1 and 8, prior to iniparib administration and 2 hours after the end of infusion.

### Treatment Administration

Iniparib was diluted in 0.9% sodium chloride to a concentration of 0.02 mg/mL to 0.2 mg/mL. All dogs received iniparib as a 60-minute IV infusion on Days 1, 4, 8, and 11. Also on Day 8, all dogs received carboplatin. CBCs were performed prior to carboplatin treatment to ensure neutrophil counts were > 1500/μL and platelet counts were > 50,000/μL permitting drug administration. Carboplatin, at a target dosage of 300 mg/m^2^ was administered immediately prior to iniparib administration via standard slow bolus injection.

A 3+3 dose escalation design was employed to allow a minimum of 3 dogs to enroll per cohort.[11] Dose escalation was performed with a goal of determining if iniparib could be safely administered at and beyond predicted exposures that are currently being used in the human clinic. Cohort escalations are denoted in Table 2. Determination of dose equivalency between dog dosing cohorts and human clinical dosing was based upon comparisons between C_max_ and AUC data collected in normal dogs and a phase I human trial.

**Table 2:** Iniparib Dose Escalation cohorts in tumor bearing dogs.

Dose Cohort	Iniparib dose	# of dogs in cohort
1	10 mg/kg (1/2 equivalent human dose)	3
2	20 mg/kg (approximate equivalent human dose)	3
3	35 mg/kg (2x equivalent human dose)	7
4	50 mg/kg (3x equivalent human dose)	3
5	70 mg/kg (4.5x equivalent human dose)	3

Toxicity due to iniparib exposure was assessable within the trial design. Toxicity was assessed using the Veterinary Cooperative Oncology Group Common Toxicity Criteria for Adverse Events v 1.1 (VCOG-CTCAE).[9] Attribution of toxicities was designated as due to drug, disease, research protocol, or other cause. Further, the certainty of attribution was designated as Unrelated, Unlikely, Possible, Probable or Definite. Dose limiting toxicities (DLT) were defined as grade 3 or higher toxicity (excluding hematologic, nausea and vomiting) refractory to standard supportive care, or grade 4 hematologic, nausea or vomiting lasting longer than 7 days or that necessitated hospitalization. Individual treatment delays or dose reductions were allowed if grade 3 or 4 adverse events occurred during the 11-day course of iniparib dosing. The maximally tolerated dose (MTD) was defined as one dose below the maximum achieved in dose escalation.

### Pharmacokinetics

Serum and plasma were collected from all dogs on Days 1, 4, 8 and 11 as outlined in Table 1. PK analysis of plasma iniparib and selected metabolites (IABA, IABM, GS-conjugate, NAC-conjugate, Cys-conjugate, and INBA) was performed on samples obtained during and after drug administration. A 12-point PK collection over 13 hours (0, 30, 55, minutes after start of infusion, 5, 15, 30 minutes, 1, 2, 4, 6, 8, 12 hours post end of infusion) was performed on Days 1 and 8 of iniparib administration. Plasma PK samples were also collected on Days 4 and 11 before treatment and at the end of infusion, and on Day 15. Serum was collected pre-treatment on Day 1, and after treatment on Days 1, 4, 8, 11, 15 and 22.

Tumor and normal tissue biopsies also were collected from all dogs to assess iniparib and selected metabolites (IABA, IABM, GS-conjugate, NAC-conjugate, Cys-conjugate, and INBA) tissue levels pre- and post therapy. Serial incisional biopsies of the tumor were collected pre-treatment (Day 1 prior to iniparib administration) and on Days 1 and 8, five minutes post administration. These biopsies were also collected on Day 15. Individual anesthesia plans were implemented by clinicians based on each dog’s history and current disease status. Normal tissue sampling from a location adjacent to the tumor (skin, mucosa or muscle) occurred concomitantly. At each biopsy time point, two samples were obtained from both the tumor and adjacent normal tissue. For tumor biopsies, one sample was flash frozen in liquid nitrogen and the other placed in formalin, whereas both normal tissue samples were flash frozen in liquid nitrogen. All biologic samples were frozen and stored at -80°C and batch shipped on Day 22 after collection for analysis using exploratory high resolution liquid chromatography coupled to mass spectrometry methods. The lower limit of quantification (LLOQ) was 2.00–5.00 ng/mL in plasma and 2.00–10.00 ng/g in tumors and normal tissues. Plasma and tissue concentrations for iniparib and metabolites on Day 1 and Day 8 were manually entered in WinNonLin 5.2 software, (Pharsight Corp., USA) using nominal sampling times, for PK analysis. Descriptive statistics (mean, standard deviation (SD), Coefficient of Variability (CV%)) were calculated on plasma and tissue concentrations per analyte and treatment group on Day 1 and Day 8.

## Results

Nineteen dogs were enrolled between March 2012 and November 2012. Dog age (median: 9 years; range: 3–14), sex (9 castrated males, 1 intact male, 7 spayed females, and 2 intact females), breed (7 mixed breeds and 12 pure breeds), and body weight (median: 27.5 kg; range: 13.8–90.2) were recorded (Table 3). One of 19 dogs was withdrawn by its owner on Day 3 after receiving only one dose of iniparib, reducing the number of evaluable dogs in Cohort 3 (35 mg/kg) from 7 to 6.

**Table 3:** Patient characteristics.

Patient	Gender	Age	Breed	Weight	Disease Term	Cohort	Response
1	Castrated male	12	Labrador Retriever	35.7 kg	Soft Tissue Sarcoma	10 mg/kg IV	SD
2	Intact female	6	Mixed Breed	31.6 kg	Soft Tissue Sarcoma	10 mg/kg IV	SD
3	Castrated male	9	Golden Retriever	45.0 kg	Fibrosarcoma	10 mg/kg IV	SD
4	Castrated male	6	Mixed Breed	22.3 kg	Soft Tissue Sarcoma	20 mg/kg IV	PD
5	Castrated male	9	Mixed Breed	23.9 kg	Soft Tissue Sarcoma	20 mg/kg IV	PD
6	Castrated male	11	Golden Retriever	24.6 kg	Soft Tissue Sarcoma	20 mg/kg IV	PD
7	Castrated male	10	German Shepherd Dog	42.1 kg	Soft Tissue Sarcoma	35 mg/kg IV	SD
8	Spayed Female	9	Mixed Breed	20.7 kg	Soft Tissue Sarcoma	35 mg/kg IV	SD
9	Castrated male	14	Mixed Breed	29.2 kg	Squamous Cell Carcinoma	35 mg/kg IV	SD
10	Spayed Female	11	Rottweiler	40.0 kg	Soft Tissue Sarcoma	35 mg/kg IV	PD
11	Castrated male	3	Great Dane	90.2 kg	Soft Tissue Sarcoma	35 mg/kg IV	SD
12	Spayed Female	8	Labrador Retriever	35.2 kg	Soft Tissue Sarcoma	35 mg/kg IV	SD
13	Intact Male	8	Vizsla	20.6 kg	Melanoma	35 mg/kg IV	SD
14	Spayed Female	12	Mixed Breed	14.9 kg	Soft Tissue Sarcoma	50 mg/kg IV	PD
15	Spayed Female	5	Labrador Retriever	27.5 kg	Squamous Cell Carcinoma	50 mg/kg IV	SD
16	Castrated male	11	Pointer	20.5 kg	Soft Tissue Sarcoma	50 mg/kg IV	SD
17	Spayed Female	11	Beagle	13.8 kg	Spindle Cell Carcinoma	70 mg/kg IV	SD
18	Female	7	Mastiff	37.2 kg	Mammary Carcinoma	70 mg/kg IV	PD
19	Spayed Female	13	Mixed Breed	17.2 kg	Soft Tissue Sarcoma	70 mg/kg IV	SD

SD: Stable Disease, PD: Progressive Disease

The number of dogs per dosing cohort is summarized in Table 2. One dog in the 35 mg/kg cohort experienced DLT including grade 3 fever, grade 4 neutropenia, and grade 4 thrombocytopenia necessitating hospitalization. These events occurred at Day 20 of the treatment cycle. This cohort was expanded and no additional DLT were observed. One dog in the 70 mg/kg cohort experienced DLT including grade 3 anorexia and grade 3 diarrhea at Day 12 of the treatment cycle, for which attribution to iniparib was deemed “Probable”. This dog also had DLT including grade 4 neutropenia and thrombocytopenia at Day 17 of the treatment cycle. This dog was hospitalized for supportive care for all of these DLT.

Iniparib was not considered the “Probable” cause of all hematologic DLT. However, all fell within the range for carboplatin neutrophil and platelet nadirs, and therefore the DLT were attributed to carboplatin alone. All other noted toxicities were mild.

Eighteen dogs were evaluable at Day 22 for tumor response assessment. There were no objective responses, complete or partial, among any of the dogs at any timepoint of study. Five of 18 (28%) had progressive disease (PD) while the remaining 13 of 18 (72%) had stable disease (SD). (Table 3)

### PK Results

Moderate to high variability was observed for iniparib and its metabolites plasma exposure levels between animals. Mean PK parameters for each dosing cohort following the 1-hour infusion of iniparib administered alone (Day 1) and with carboplatin (Day 8) are presented in Table 4. Following a 1-hour infusion on Day 1, all animals were exposed to iniparib and its metabolites. Detectable concentrations were above LLOQ up to 2.0 hours, up to 3.0 hours and up to 13.0 hours after the start of the 1-hour infusion for iniparib, for IABM, and for all other metabolites, respectively regardless of dosage administered. The main quantifiable metabolites in plasma with highest exposures, regardless of the dose and the day administered were GS-conjugate, Cys-conjugate, and INBA. Across all dosing cohorts (10–70 mg/kg), iniparib and its metabolites exposures (C_max_ and AUC) were roughly similar regardless of whether iniparib was administered alone (Day 1) or in combination with carboplatin (Day 8).

**Table 4:** Pharmacokinetics and tissue biodistribution of iniparib and selected metabolites in the tumor-bearing dog.

Compound	Dose (mg/kg)	Day	Plasma Cmax (ng/ml)	Plasma AUC (ng*hr/mL)	Plasma T1/2z (hr)	Ratio Tumor/Plasma
Iniparib	10	1	1340 ± 659	1100**	0.092**	NA
	20	1	3540 ± 2870	2800 ± 2200	0.13 ± 0.037	0.074
	35	1	12400 ± 11100	8000 ± 8200	0.11 ± 0.021	0.073
	50	1	11000 ± 9820	9100**	0.13**	NA
	70	1	24900 ± 19300	9000**	0.11**	0.088
Iniparib	10	8	1620 ± 712	1100 ± 260	0.1 ± 0.013	NA
	20	8	3100 ± 1340	2000 ± 800	0.16 ± 0.076	NA
	35	8	9080 ± 6150	4300 ± 1300	0.12 ± 0.018	0.057
	50	8	11600 ± 11500	NA	NA	NA
	70	8	10500 ± 6870	6400 ± 3700	0.17 ± 0.051	NA
IABM	10	1	7.02 ± 1.84	NA	NA	NA
	20	1	10 ± 1.35	NA	NA	NA
	35	1	29.6 ± 18.2	67 ± 37	0.78 ± 0.14	1.4
	50	1	66.6 ± 32.2	97 ± 31	0.91 ± 0.29	NA
	70	1	36.3 ± 11.9	NA	NA	NA
IABM	10	8	6.96**	NA	NA	NA
	20	8	12.8 ± 1.45	NA	NA	NA
	35	8	30 ± 14.1	75**	1**	1.7
	50	8	37.7 ± 16.8	66**	0.66**	NA
	70	8	86.6 ± 73.1	NA*	NA*	NA
IABA	10	1	10.9 ± 5.48	94**	2.5**	NA
	20	1	16.8 ± 6.61	81±29	2 0.23	NA
	35	1	71.7 ± 30	390 ± 180	2.3 0.7	NA
	50	1	96.5 ± 27.1	510 ± 170	2.1 0.15	NA
	70	1	116 ± 42.4	750 ± 230	2.5 0.57	0.49
IABA	10	8	7.29 ± 4.1	63**	2.7**	NA
	20	8	13.9 ± 3.11	NA	NA	NA
	35	8	57 ± 22.3	330 ± 210	2.2 ± 0.82	1
	50	8	56.4 ± 20.7	270 ± 95	1.9 ± 1.2	NA
	70	8	149 ± 56	920 ± 200	2.5 ± 0.9	NA
Glutathione-conjugate	10	1	7020 ± 1370	9200 ± 2100	5.6 ± 3.7	0.52
	20	1	13100 ± 4710	18000 ± 5800	6.7 ± 5.2	0.18
	35	1	27000 ± 5680	36000 ± 8200	4 ± 2.2	0.28
	50	1	55800 ± 9150	73000 ± 14000	2 ± 0.59	0.23
	70	1	42500 ± 29800	69000**	2.6**	NA
Glutathione-conjugate	10	8	7060 ± 544	9400 ± 1400	2.2 ± 0.12	0.4
	20	8	12100 ± 4100	16000 ± 4600	3.2 ± 0.99	0.071
	35	8	28100 ± 7050	39000 ± 9100	2.5 ± 0.46	0.56
	50	8	41800 ± 3860	64000 ± 7400	2.8 ± 1.9	0.37
	70	8	39400 ± 33400	64000 ± 48000	3.1 ± 0.4	NA
N-acetyl-cysteine-conjugate	10	1	14.7**	NA	NA	NA
	20	1	31.3 ± 3.7	NA	NA	NA
	35	1	48.3 ± 28.1	290**	3.2**	NA
	50	1	77.8 ± 21.4	430**	2.2**	NA
	70	1	92.8 ± 14.2	670**	4.1**	NA
N-acetyl-cysteine-conjugate	10	8	11.8 ± 6.71	NA	NA	NA
	20	8	34.2 ± 4.15	NA	NA	NA
	35	8	45.9 ± 13.3	220**	2**	NA
	50	8	98.1 ± 21.7	480 ± 290	2.6 ± 0.91	NA
	70	8	93.8 ± 63.4	NA	NA	NA
Cysteine-conjugate	10	1	5740 ± 883	15000 ± 3500	3.3 ± 1.8	1.2
	20	1	7880 ± 1050	22000**	3.1**	0.6
	35	1	16800 ± 4280	42000 ± 17000	3.5 ± 1.2	0.84
	50	1	24600 ± 1610	63000 ± 21000	2.3 ± 0.89	0.37
	70	1	21300 ± 5370	68000**	2.4**	0.9
Cysteine-conjugate	10	8	5910 ± 648	12000 ± 2100	1.9 ± 0.23	0.72
	20	8	7540 ± 1010	19000 ± 3000	2.6 ± 1.1	1.4
	35	8	15700 ± 4250	40000 ± 12000	2.5 ± 0.47	0.75
	50	8	22300 ± 4720	64000 ± 19000	2.9 ± 1.8	0.52
	70	8	23100 ± 9680	63000 ± 25000	2.9 ± 0.6	0.54
INBA	10	1	6200 ± 1510	11000 ± 3900	1.2 ± 0.5	0.29
	20	1	16200 ± 4950	27000 ± 4200	1.3 ± 0.2	0.16
	35	1	32200 ± 7750	62000 ± 18000	1.3 ± 0.095	0.12
	50	1	36400 ± 18000	68000 ± 24000	1.3 ± 0.15	0.16
	70	1	55600 ± 15900	150000 ± 31000	1.2 ± 0.058	0.25
INBA	10	8	6280 ± 795	9800 ± 2300	0.82 ± 0.18	0.31
	20	8	10500 ± 2250	19000 ± 3800	1.5 ± 0.36	0.3
	35	8	29700 ± 6100	57000 ±16000	1.3 ± 0.2	0.16
	50	8	30700 ± 16200	54000 ± 22000	1.6 ± 0.44	0.17
	70	8	47300 ± 8860	120000 ± 27000	1.2 ± 0.058	0.15

NA = not assessed or not calculable as below the Lower Limit of Quantification (LLOQ) for sample type

Data presented as mean +/- standard deviation

* n = 1;

** n = 2

As observed in plasma, the main quantifiable metabolites in normal and tumor tissues were GS-conjugate, Cys-conjugate, and INBA; these were quantifiable at all iniparib doses. Normal and tumor tissue exposure of iniparib and IABA was quantifiable at lower levels, but not at all doses or in all dogs, thus precluding the calculation of tumor:normal tissue ratios. IABM was also quantifiable in tumors at lower levels but was distributed with the highest tumor/plasma ratios assessed at 1.4 and 1.7 on Day 1 and Day 8, respectively at the dose of 35 mg/kg of iniparib. Tumor to plasma ratios for iniparib and metabolites for each dosing cohort following the 1-hour infusion of iniparib administered alone (Day 1) and with carboplatin (Day 8) are also presented in Table 4.

## Discussion

The value of the comparative oncology model, using pet dogs with spontaneous cancers to study novel cancer therapies, has been previously described and illustrates the complementary advantages to the approach.[12,13] Key features of this model system are that it allows investigations to occur in immune-competent, large animals (pet dogs) with naturally occurring tumors, which confers advantages such as the presence of naturally syngeneic tumor-associated vasculature and stroma, tumor heterogeneity, and host immune responses. One particular challenge of the traditional rodent model that the pet dog model overcomes is the ability to collect repeated blood and tissue samples to study drug PK. The large size of dogs makes such sample collection feasible. Additionally, as is the case with iniparib, the PK and metabolism of drugs is often more comparable between humans and dogs in contrast to other conventional preclinical models. Finally, many common canine cancers are comparable to common human cancers in etiology, biologic behavior, response to therapy, and overall outcome.[14]

Results of this study demonstrate that iniparib can be administered safely in combination with carboplatin to dogs with naturally occurring cancers at and above dosages relevant to those used in the human clinic. When carboplatin is added to the treatment regime, toxicity does not increase beyond that expected with carboplatin alone. Iniparib is cleared rapidly from plasma of tumor-bearing dogs over the dose range of 10–70 mg/kg. However, at dosages up to 70 mg/kg neither iniparib nor its metabolites accumulate significantly in tumor or normal tissues. PK and tolerability data collected in human patients receiving iniparib alone and in combination with gemcitabine/carboplatin chemotherapy revealed striking similarities to data reported herein.[3,4] Although a high degree of variability was seen, these human data collected from patients receiving 5.6 mg/kg of iniparib twice weekly are comparable to the tumor-bearing dog dosing cohorts of 10–20 mg/kg of iniparib alone and in combination with carboplatin. These results are consistent with previous studies that demonstrate the tolerability but lackluster performance of iniparib in human clinical trials.[4] Recent studies suggest that clinical benefit could be seen with inparib in combination with temozolomide, and when given at doses significantly higher than previously studied. The dog model provides an excellent opportunity to delve further into these observations by virtue of the facile nature of serial biologic sample collections within individual canine patients and existing clinical expertise among veterinary oncologists with such treatment strategies. The data presented herein provides a natural platform on which to base such future studies of the relationship between PK, PD and drug combinations. Furthermore, these data provide significant support to the use of the canine model, particularly when comparative PK is critical to determining if drug target modulation occurs at tolerable doses with meaningful accumulation within tumor tissue. Indeed, the lack of robust understanding of drugs’ PK-PD relationships, target modulation, and predictive biomarkers are cited as key factors in oncology drug development attrition.[15,16]

Comparative oncology trials often precede or are conducted in parallel with human trials, and in many cases guide the development of such trials.[17,18] This particular comparative oncology study with iniparib was performed in parallel with iniparib trials in humans. Although development of iniparib as an anticancer agent has been terminated, this study demonstrates the potential for the comparative approach to add new data to the totality of drug development (human and conventional preclinical models) to assist in clarifying PK, PD and combination tolerability of investigative drugs, all of which are needed for successful drug development.

In conclusion, this comparative oncology study in tumor-bearing pet dogs confirms that the PK properties and clinical tolerability of iniparib mirror what is observed in human cancer patients, both as a single agent and in combination with other chemotherapies. The tissue biodistribution data confirms iniparib’s inability to reach relevant tissue levels. This, combined with knowledge that PARP inhibition is not iniparib’s main mechanism of action may account for the lackluster results in confirmatory human TNBC clinical trials.

## Supporting Information

S1 Poster Pharmacokientics and metabolism of iniparib for the treatement of metastatic triple-negative breast cancer. (PDF)
