# Supplementary material for: A Comparative Oncology Study of Iniparib Defines Its Pharmacokinetic Profile and Biological Activity in a Naturally-Occurring Canine Cancer Model
Source: PLoS One. 2016 Feb 11;11(2):e0149194. doi: 10.1371/journal.pone.0149194 (PMC4751284; doi:10.1371/journal.pone.0149194)
Supplement: S1 Poster — (PDF) [file pone.0149194.s001.pdf]

A134

# PHARMACOKINETICS AND METABOLISM OF INIPARIB FOR THE TREATMENT OF METASTATIC TRIPLE-NEGATIVE BREAST CANCER (TNBC)


Jaap Verweij,[1] Veronique Diéras,[2] Kevin Rockich,[3] Yaming Su,[3] Dominique Mery-Mignard,[4] Nga Pham,[4] and Gary Emmons[3]

[1]Department of Medical Oncology, Erasmus University Medical Center, Rotterdam, Netherlands; [2]Department of Medical Oncology, Institut Curie, Paris, France; [3]Sanofi, Bridgewater, NJ; [4]Sanofi, Vitry sur Seine, Paris, France


## Introduction

- Iniparib (BSI-201, SAR240550, 4-iodo-3-nitrobenzamide) is an investigational anticancer agent.
- In breast cancer cell lines and xenograft models of triple-negative breast cancer (TNBC), iniparib has antiproliferative activity and potentiates the cell cycle effects of some DNA damaging agents.[1–3]
- In a randomized, open-label Phase 2 study in patients with metastatic TNBC (mTNBC), iniparib combined with gemcitabine (G) and carboplatin (C) improved efficacy outcomes compared with GC alone.[4]
- In a confirmatory Phase 3 study, treatment with GC + iniparib failed to meet pre-specified criteria for improvement in progression-free survival and overall survival; however, an exploratory subset analysis suggested a potential benefit among second- and third-line patients.[5]
- Here we report on the pharmacokinetics (PK) and metabolism of iniparib in patients enrolled in two clinical studies:
  - Phase 1 [$^{14}$C] absorption, distribution, metabolism and excretion (ADME) study of iniparib in patients with solid tumors (BEX11505; NCT01161836)
  - Phase 2 study of iniparib administered twice-weekly or once-weekly in combination with GC in patients with mTNBC (TCD11418; NCT01045304).

## Methods

### Schema 1. BEX11505 Phase 1 study design

**BEX11505 Study Population (N=7)**
- Advanced solid tumors refractory to standard treatment or no standard treatment exists

- [$^{14}$C]-iniparib 400 mg
- Single dose IV over 60 minutes; average dose 5.22 mg/kg (CV14.8%)
- Followed by extended treatment with iniparib with or without chemotherapy

**Study Objectives**

Primary endpoints
- Exposure and excretion of [$^{14}$C]-iniparib
- Pharmacokinetics
- QT/QTc interval changes
- Identification of iniparib metabolites

Secondary endpoint
- Tolerability

CV, coefficient of variation

- Plasma, urine and fecal samples were collected for 10 days for:
  - Measurement of iniparib, and the metabolites 4-iodo-3-aminobenzamide (IABM) and 4-iodo-3-aminobenzoic acid (IABA) from plasma with a validated liquid chromatography-tandem mass spectrometry (LC/MS-MS)
  - Measurement of plasma, urine and feces $^{14}$C by liquid scintillation counting
  - Metabolic profiling and identification of metabolites in plasma, urine and feces by radio-LC and LC/MS (LTQ-Orbitrap).

### Schema 2. TCD11418 Phase 2 study design

**TCD11418 Study Population (N=163)**
- Stage IV mTNBC
- ≤2 prior chemotherapy regimens for metastatic disease
- Stratified 0 vs. 1–2 prior regimens

**Randomization (1:1)**

- Iniparib 11.2 mg/kg weekly schedule
- Gemcitabine 1000 mg/m$^2$
- Carboplatin AUC2
- 21-Day cycle

n=82

- Iniparib 5.6 mg/kg twice-weekly schedule
- Gemcitabine 1000 mg/m$^2$
- Carboplatin AUC2
- 21-Day cycle

n=81

**Study Objectives**

Primary endpoint
- Overall response rate (ORR)

Secondary endpoints
- Clinical benefit rate (CBR = CR + PR + SD ≥24 weeks)
- Progression-free survival (PFS)
- Overall survival (OS)

CR, complete response; PR, partial response; SD, stable disease

- Iniparib was administered as a 60-minute intravenous infusion to female patients with mTNBC in combination with GC and continued until disease progression, unacceptable toxicity or withdrawal of consent (no maximum number of cycles).
- Randomization was to treatment with iniparib, either once-weekly (11.2 mg/kg) on Days 1 and 8, or twice-weekly (2 x 5.6 mg/kg) on Days 1, 4, 8 and 11.
- Plasma was collected pre- and post-iniparib dose during Cycle 1 on Days 1, 8 and 11 for PK analysis of iniparib, IABM and IABA by a validated LC/MS-MS method.

## Results

- PK/metabolism data were available from:
  - All 7 patients (2 male, 5 female) in study BEX11505
  - 69 out of 163 patients (42%) receiving iniparib either twice-weekly (N=34) or once-weekly (N=35) in the TCD11418 trial.
- In both studies, the safety profile was consistent with results of previous studies of iniparib as a single-agent and for the GC + iniparib combination.[4,6]

**Table 1. Individual and mean excretion balance, and total recovery of radioactivity (% of iniparib dose) from patients in the BEX11505 study (N=7)**

| | Patient Number | | | | | | | | |
|---|---|---|---|---|---|---|---|---|---|
| | 1 | 2 | 3 | 4 | 5 | 6 | 7 | Mean | SD |
| **Urine** | 73.8 | 67.7 | 72.1 | 70.7 | 75.4 | 72.2 | 76.4 | 72.6 | 2.94 |
| **Feces** | 3.61 | 22.7 | 18.8 | 17.6 | 13.5 | 19.7 | 16.0 | 16.0 | 6.18 |
| **Total** | 77.4 | 90.4 | 90.9 | 88.3 | 88.9 | 91.9 | 92.4 | 88.6 | 5.15 |

**Figure 1. Mean (± SD) iniparib, IABM, and IABA, concentrations in plasma* from patients in the BEX11505 study (N=7)**

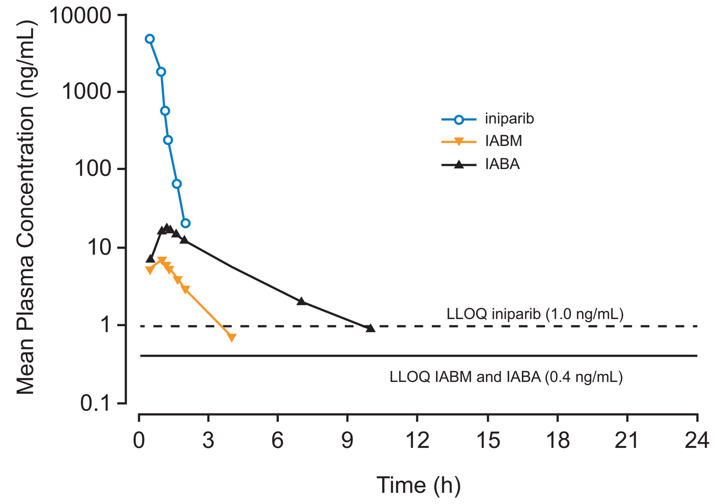


* Blood was collected from a central line for iniparib/IABM/IABA measurements; IABA, 4-iodo-3-aminobenzoic acid; IABM, 4-iodo-3-aminobenzamide; IHABM, 4-iodo-3-hydroxyaminobenzamide; LLOQ, lower limit of quantitation

**Figure 2. Mean (± SD) iniparib and total [$^{14}$C] radioactivity concentrations in plasma or whole blood from patients in BEX11505 (N=7)**

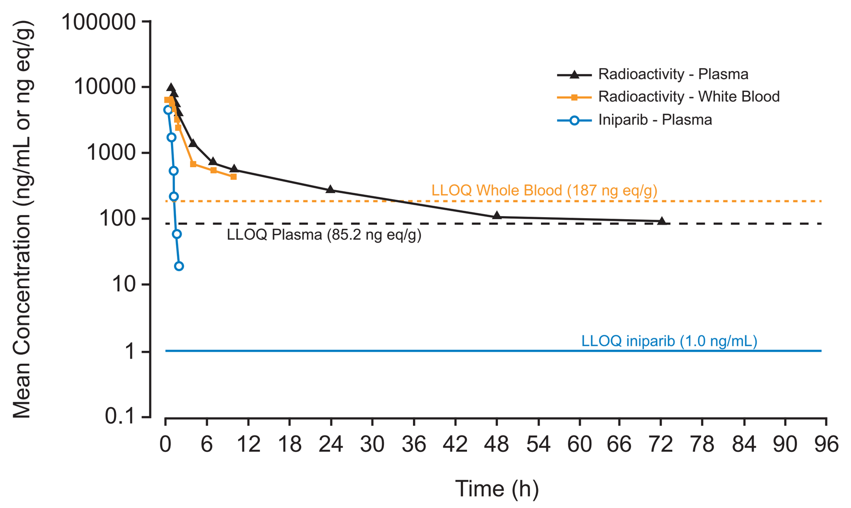


LLOQ, lower limit of quantitation

**Figure 3. Typical radiochromatogram of plasma AUC (generally a zero to 24 h pool) following a single intravenous infusion of [$^{14}$C]-iniparib at 400 mg (BEX11505)**

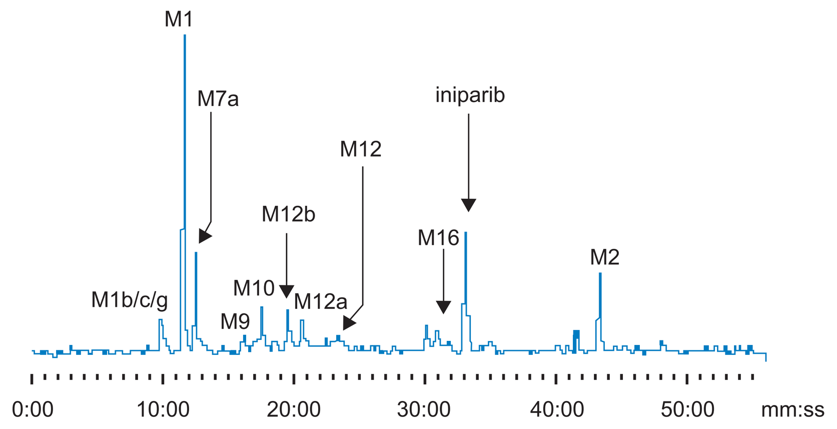


- M1, M7a and M12 are further metabolites of the glutathione adduct of iniparib (M9).
- M2 is the metabolite from the amide hydrolysis of iniparib.
- Neither IABM nor IABA were detected by measuring radioactivity, but they were detected by LC/MS-MS.

**Figure 4. Typical radiochromatogram of urinary radioactivity (95% pool) following a single intravenous infusion of [$^{14}$C]-iniparib at 400 mg (BEX11505)**

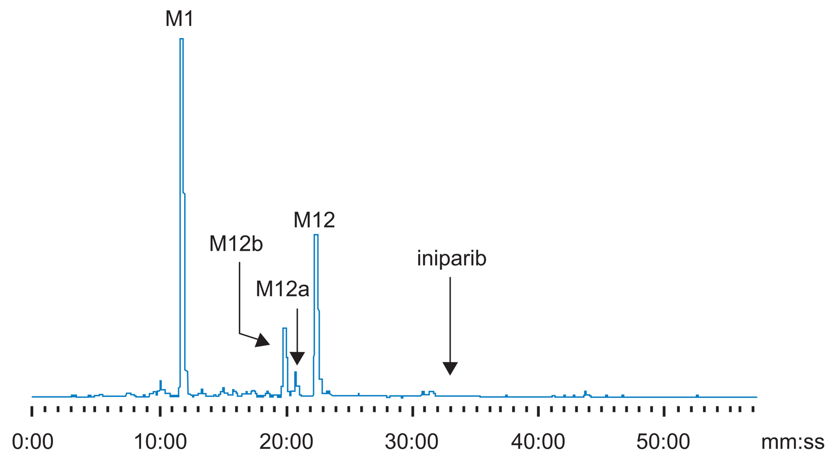


- No unchanged iniparib was detected in urine.
- M1, M12 and M12a are further metabolites of the glutathione adduct of iniparib.

**Figure 5. Typical radiochromatogram of fecal radioactivity (95% pool) following a single intravenous infusion of 400 mg [$^{14}$C]-iniparib (BEX11505)**

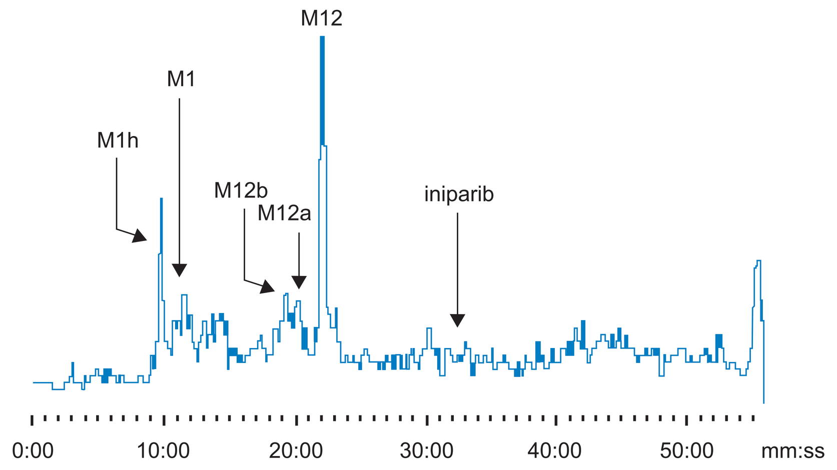


- Radiochromatograms of fecal radioactivity were qualitatively similar but quantitatively variable from patient to patient.
- No iniparib, IABM or IABA were detected in any samples.
- M1, M1h, M12 and M12a are further metabolites of a glutathione adduct of iniparib.

**Table 2. Iniparib pharmacokinetics (TCD11418)**

| | | | Iniparib, mean (CV%) | | | |
|---|---|---|---|---|---|---|
| Schedule | Day | n | $t_{max}$ (h) | $C_{max}$ (ng/mL) | AUC (ng.h/mL) | $t_{1/2}$ (h) |
| **Twice-weekly** | 1 | 34 | 0.92 | 2190 (42%) | 2060 (34%) | 0.65 (146%) |
| (5.6 mg/kg) | 11 | 32 | 0.92 | 2410 (56%) | 2420 (68%) | 1.4 (139%) |
| **Once-weekly** | 1 | 34 | 0.69 | 3820 (41%) | 4160 (119%) | 0.92 (136%) |
| (11.2 mg/kg) | 8 | 33 | 0.58 | 3790 (44%) | 3610 (36%) | 1.32 (121%) |

Iniparib PK data were not available for 1 patient; CV, coefficient of variation'

- Iniparib exposure increased approximately 2-fold with the 2-fold higher dose of 11.2 mg/kg compared with the 5.6 mg/kg dose.
- There was no accumulation in exposure on either Days 8 or 11.
- Maximum plasma concentrations of iniparib were generally reached between 30 and 60 minutes after the start of the infusion.

**Table 3. IABM and IABA pharmacokinetics (TCD11418)**

| | | | IABM, mean (CV%) | | | | IABA, mean (CV%) | | | |
|---|---|---|---|---|---|---|---|---|---|---|
| Schedule | Day | n | $t_{max}$ (h) | $C_{max}$ (ng/mL) | AUC (ng.h/mL) | $t_{1/2}$ (h) | $t_{max}$ (h) | $C_{max}$ (ng/mL) | AUC (ng.h/mL) | $t_{1/2}$ (h) |
| **Twice-weekly** | 1 | 34 | 1 | 7.5 (49%) | 14.1 (49%) | 0.82 (26%) | 1.33 | 13.1 (63%) | 38.9 (52%) | 1.8 (22%) |
| (5.6 mg/kg) | 11 | 32 | 1 | 8.3 (51%) | 15.9 (40%) | 0.98 (33%) | 1.32 | 13.2 (58%) | 41.4 (51%) | 1.91 (36%) |
| **Once-weekly** | 1 | 35 | 1 | 16.0 (47%) | 27.6 (32%) | 0.84 (31%) | 1.33 | 30.9 (54%) | 92.5 (62%) | 1.81 (21%) |
| (11.2 mg/kg) | 8 | 33 | 1 | 15.9 (46%) | 27.3 (37%) | 0.85 (21%) | 1.33 | 31.8 (44%) | 91.6 (43%) | 1.84 (25%) |

CV, coefficient of variation

- Iniparib metabolites had slightly longer half-lives than iniparib.
- Maximal concentrations of the metabolites occurred at or near the end of the infusion.
- Concentrations were only a fraction of those seen for iniparib.

**Figure 6. Plasma PK analysis from study TCD11418: Blood was sampled on Day 1 of Cycle 1 from patients on a twice-weekly or once-weekly schedule of iniparib**

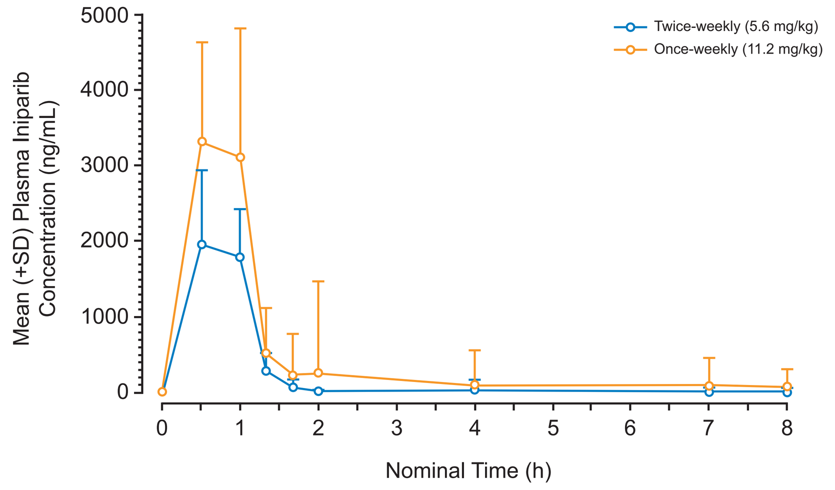


**Figure 7. Proposed metabolic pathways for iniparib**

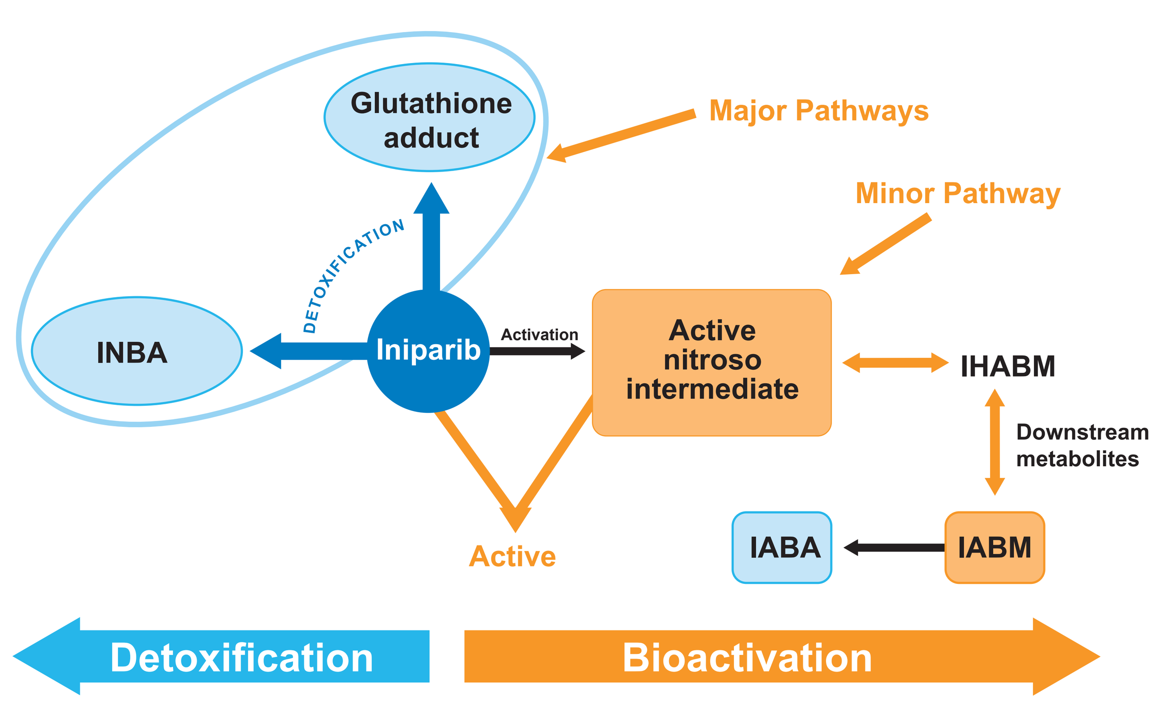


IABA, 4-iodo-3-aminobenzoic acid; IABM, 4-iodo-3-aminobenzamide; IHABM, 4-iodo-3-hydroxyaminobenzamide; INBA, 4-iodo-3-nitrobenzoic acid

## Conclusions

- Iniparib is rapidly distributed throughout the body and quickly metabolized.
- Clearance from plasma is rapid with a half-life of 10–20 minutes, but the half-life of total $^{14}$C-labeled metabolites in plasma is approximately 18.5 hours.
- Much of the drug is converted to inactive glutathione adducts, with the glutathione moiety undergoing further metabolism.
- Another significant metabolic pathway leading to deactivation of the drug involves hydrolysis of the amide function.
- Evidence for biotransformation to the nitroso metabolite via nitro-reduction was indicated by the detection of IABM and IABA in plasma, however their concentrations were less than 2% of that of unchanged drug.
- More recent *in vitro* studies have demonstrated the formation of the highly unstable metabolite, 4-iodo-3-hydroxyaminobenzamide (IHABM).
- Iniparib, IABM and IABA exposure ($C_{max}$ and AUC) increased in mTNBC patients approximately proportionally when the dose was increased from 5.6 mg/kg to 11.2 mg/kg.
- No accumulation of iniparib, IABM or IABA was observed with either once-weekly or twice-weekly doing.


## Acknowledgments

- Editorial assistance was provided by ArticulateScience Ltd and was supported by Sanofi.


## References


1. Ossovskaya V, *et al. Proc Amer Assoc Cancer Res* 2009;Abstr 5552.
2. Ossovskaya V, *et al. Proceedings of the San Antonio Breast Cancer Symposium* 2010;Abstr P5-06-09.
3. Ossovskaya V, *et al. Proc Amer Assoc Cancer Res* 2011;Abstr LB-401.
4. O'Shaughnessy J, *et al. N Engl J Med* 2011;364:205–214.
5. O'Shaughnessy J, *et al. J Clin Oncol (Suppl)* 2011;29:Abstr 1007.
6. Kopetz S, *et al. Proc Amer Assoc Cancer Res* 2011;Abstr 2311.



Presented at the AACR-NCI-EORTC meeting, November 12–16, 2011, San Francisco, CA, USA
